# Chronic contact with realistic soil concentrations of imidacloprid affects the mass, immature development speed, and adult longevity of solitary bees

**DOI:** 10.1038/s41598-019-40031-9

**Published:** 2019-03-06

**Authors:** Nicholas L. Anderson, Alexandra N. Harmon-Threatt

**Affiliations:** University of Illinois at Urbana-Champaign, Department Of Entomology, 505 S. Goodwin Ave., Urbana, IL 61801 United States

## Abstract

The non-target effects of pesticides are an area of growing concern, particularly for ecologically and economically important organisms such as bees. Much of the previous research on the effects of neonicotinoids, a class of insecticide that has gained attention for non-target effects, on bees focused on the consumption of contaminated food resources by a limited number of eusocial species. However, neonicotinoids are known to accumulate and persist in soils at concentrations 2 to 60 times greater than in food resources, and may represent an important route of exposure for diverse and ecologically important ground-nesting bees. This study aimed to assess the effect of chronic contact exposure to realistic soil concentrations of imidacloprid, the most widely used neonicotinoid pesticide, on bee longevity, development speed, and body mass. Cohorts of *Osmia lignaria* and *Megachile rotundata* were used as proxies for ground-nesting species. We observed species- and sex-specific changes to adult longevity, development speed, and mass in response to increasing concentrations of imidacloprid. These results suggest that chronic exposure to nesting substrates contaminated with neonicotinoids may represent an important route of exposure that could have considerable physiological and ecological consequences for bees and plant-pollinator interactions.

## Introduction

For much of the past two decades, research on the lethal (e.g., increased mortality over 24–48 hours) and sublethal (e.g., reduced performance) non-target effects of neonicotinoids on pollinators has primarily focused on the consumption of contaminated pollen and nectar in honeybees and, more recently, bumblebees^1^. Although there appears to be no consistent effect on adult mortality rates in honeybees at dosages commonly recovered from pollen and nectar, a wide range of significant sublethal effects of acute and chronic exposure are well documented^2^. Observed sublethal effects include delayed larval development^3^, impaired mushroom body growth and neurological function^4–6^, and disruptions to reproduction including reduced production of reproductive female offspring^7–10^. While the consumption of neonicotinoids by honeybees and bumblebees may have important economic and ecological implications, there is also a need to assess additional routes of exposure and bee species to gain a better understanding of the non-target effects of neonicotinoids on bee communities as a whole.

With most bees nesting underground^11^, prolonged contact with neonicotinoid contaminated soils may represent a significant route of exposure for many species. However, field-scale assessments of the effects of neonicotinoids on native bees have largely ignored the potential effects of contaminated nesting resources even when a number of affected species are ground-nesting and not thought to collect food resources from treated plants, e.g.^12^. While lethal concentrations of neonicotinoids are higher for contact than oral exposure^13^, soil concentrations of neonicotinoids often reach higher and more persistent levels than those in pollen and nectar. Soil concentrations of imidacloprid, a commonly used neonicotinoid, are often between 12–18 ppb, with values of up to 650 ppb reported, compared to 1–11 ppb in pollen and nectar^14–18^. Soils become contaminated with high concentrations of neonicotinoids as a result of much of the active ingredient, commonly applied as a seed treatment, spreading into the surrounding soil rather than being absorbed by targeted plants^16,19^, returning to the soil as treated plant material decomposes^20^, and having a relatively long half-life in soils^14,21–23^. Additionally, the long immature development period, relative to adult lifespan, exhibited by ground-nesting bees may amplify the effects of contaminated soils as the toxicity of neonicotinoids increases with exposure time^24,25^. The lack of an assessment of the effects of chronic contact exposure to realistic soil levels of neonicotinoids represents a major gap in our current knowledge, especially given the number of species at risk.

Using imidacloprid, the most well-studied member of the neonicotinoid insecticide family^24,26^, we evaluated the sublethal effects of chronic contact exposure to realistic soil concentrations during immature development on *Osmia lignaria* Say, 1837 and *Megachile rotundata* (Fabricius, 1787). While not ground-nesting species themselves, *O*. *lignaria* and *M*. *rotundata* belong to genera containing ground-nesting species and were used previously to approximate the effects of soil conditions on soil-dwelling species^27^. The benefits to employing these species as proxies for ground-nesting bees are that they are easy to collect and rear and represent a worst case scenario of soil contact without a nest cell lining - structures that are highly variable within and between taxa^28–33^.

We hypothesised that chronic contact exposure to imidacloprid would disrupt normal bee physiological functioning, possibly by altering the expression of genes associated with metabolism or detoxification^34–36^ or reducing motor function^5^. These changes were expected to manifest as a decrease in body mass, development speed, or immature or adult longevity which could affect populations by reducing the total number of nest cells provisioned or altering emergence timing which disrupts mating and flower visitation. Due to differences in body mass (Table 1a), life histories (Table 1b), and the number of chromosomes (i.e., haplodiploid sex determination), we predicted that observed effects would be stronger for *M*. *rotundata* and male bees when compared to *O*. *lignaria* and female bees, respectively.

**Table 1:** Differences in the ecology of and the methodologies used for *Osmia lignaria* and *Megachile rotundata*.

	*Osmia lignaria*	*Megachile rotundata*
(a) Average adult mass ± 95% C.I. (mg)	Female: 98.57 ± 4.55	Female: 27.65 ± 1.49
	Male: 52.03 ± 2.42	Male: 23.97 ± 3.48
(b) Overwintering stage	Pre-emergent adult	Prepupa
(c) Tissue culture plates used	24 well	Summer 2015 - Spring 2016: 96 well
		Summer 2016: 24 well
(d) Nesting substrates provided	8 mm diameter paper straws	6 and 8 mm diameter paper straws
	Clay-soil mixture	Assorted leafy plants
(e) Number of imidacloprid applications	2015: 62	2015: 26
	2016: 0	2016: 9
	Total: 62	Total: 35
(f) Cumulative imidacloprid dose (ng active ingredient bee^−1^)	0 ppb: 0	0 ppb: 0
	7.5 ppb: 0.233	7.5 ppb: 0.131
	15 ppb: 0.465	15 ppb: 0.263
	100 ppb: 3.1	100 ppb: 1.75

Differences in body size and life history traits of *O*. *lignaria* and *M*. *rotundata* led to practical differences in the methods used for each species.

## Results

The effects of chronic contact exposure to realistic soil concentrations of imidacloprid during immature development varied between *O*. *lignaria* and *M*. *rotundata* and often between males and females of the same species.

**Figure 1:**
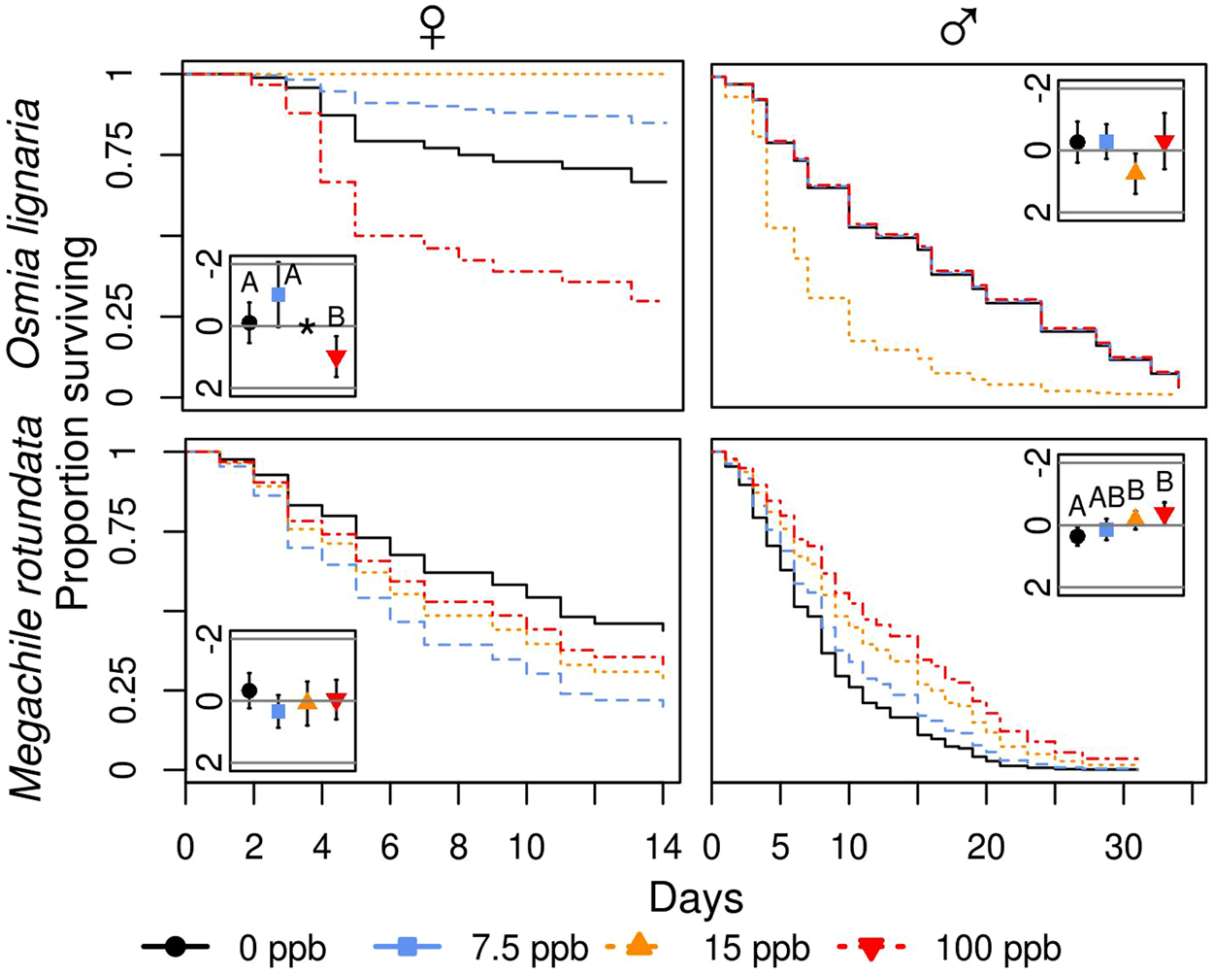
Effect of realistic soil concentrations of imidacloprid on adult bee longevity. Survival curves represent the proportion of bees that were alive on a given day. Inset graphs display the log hazard ratios ±95% confidence intervals (y-axis) associated with each imidacloprid treatment level (x-axis). Values below the centre line (i.e. more positive) represent a higher probability that an individual will die on a given day, provided that it has not already done so, relative to the overall mean. Values above the line indicate the opposite. Capital letters are used to signify significant differences (P < 0.05). *No adult female *O*. *lignaria* in the 15 ppb group (n = 16) died before being censored for a concurrent project, and a survival curve and hazard ratios cannot be estimated.

In *O*. *lignaria*, we only detected an effect on adult female longevity which had an inverted u-shape, with a slight increase in longevity at low concentrations of imidacloprid and a decrease at high concentrations (Figs 1 and S1–S3; Tables 2–4). Individuals treated with the highest concentration, 100 ppb, lived an average of 4.5 and 5 days fewer than 0 and 7.5 ppb treated bees (P = 0.032, P = 0.011, respectively). However, it is possible that these effect sizes are underestimated as most female *O*. *lignaria* in the 0 and 7.5 ppb groups survived until 14 days after emergence when they were censored for use in a concurrent study. Additionally, no *O*. *lignaria* females treated with the 15 ppb imidacloprid solution died before being censored (n = 16), and, thus, we were not able to fit a survival function for this group or statistically compare them to the other treatment levels. Despite this, we interpret the 15 ppb female *O*. *lignaria* as having lived longer than their 100 ppb treated counterparts and that there is a potential trend for increased longevity over individuals in the control group. Effects on male *O*. *lignaria* adult longevity and mass are potentially obscured by a loss of statistical power caused by an equipment malfunction during the overwintering period resulting in the loss of 70% (66 individuals) of adult male bees from across all treatments (Table S1). Therefore, we advise caution when interpreting the result of no detected effects on male *O*. *lignaria* adult longevity and mass.

**Table 2:** Summary of Cox Proportional-Hazards Regression models for bee longevity.

Species	Life stage/sex	d.f.	Wald χ^2^	P-value
*Osmia lignaria*	Pooled immature	3	1.4	0.705
	Adult female	3	8.62	0.013*
	Adult male	3	5.14	0.162
*Megachile rotundata*	Pooled immature	3	4.1	0.251
	Adult female	3	2.23	0.527
	Adult male	3	8.52	0.036*

*Significant at the 0.05 level.

^+^Significant at the 0.10 level.

**Table 3:** Summary of the Prentice, Williams, and Peterson total time extension for multiple events of the Cox Proportional-Hazards Regression models for bee development speed.

Species	Sex	Effect	d.f.	Pre-overwintering stages		Post-overwintering stages	
				Wald χ^2^	P-Value	Wald χ^2^	P-Value
*Osmia lignaria*	Female	Imidacloprid	3	5.07	0.167	2.03	0.567
		Treatment start date	1	61.95	<0.001*	11.75	0.001*
	Male	Imidacloprid	3	0.58	0.902	0.59	0.899
		Treatment start date	1	66.43	<0.001*	25.98	<0.001*
*Megachile rotundata*	Female	Imidacloprid	3	7.29	0.063^+^	6.70	0.082^+^
		Treatment start date	1	—^#^	—^#^	28.86	<0.001*
	Male	Imidacloprid	3	0.91	0.823	8.82	0.032*
		Treatment start date	1	3.67	0.055^+^	19.29	<0.001*

^*^Significant at the 0.05 level.

^+^Significant at the 0.10 level.

^#^Treatment start date, an estimate of when eggs were laid, was determined not to be significant for *M*. *rotundata* female pre-overwintering development speed and was removed from the model (χ^2^_1_ = 0.01, 1 df, P = 0.9277).

**Table 4:** Summary of mixed-effects models for bee mass.

Species	Sex	Effect	d.f.	F	P-value
*Osmia lignaria*	Female	Imidacloprid	3,102	1.11	0.350
		Development stage	3,142	463.84	<0.001*
		Imidacloprid * Development stage	9,194	1.28	0.251
		Natal nest cell mass	1,110	307.12	<0.001*
	Male	Imidacloprid	3,133	0.44	0.723
		Development stage	3,140	314.11	<0.001*
		Imidacloprid * Development stage	3,185	0.41	0.928
		Natal nest cell mass	1,140	43.67	<0.001*
*Megachile rotundata*	Female	Imidacloprid	3,58.7	2.49	0.069^+^
		Development stage	3,75.3	260.80	<0.001*
		Imidacloprid * Development stage	9,106	1.20	0.301
		Natal nest cell mass	1,66.4	9.01	0.004*
	Male	Imidacloprid	3,107	2.92	0.038*
		Development stage	3,151	825.83	<0.001*
		Imidacloprid * Development stage	9,212	0.97	0.468
		Natal nest cell mass	1,120	11.30	0.001*

*Significant at the 0.05 level.

^+^Significant at the 0.10 level.

**Figure 2:**
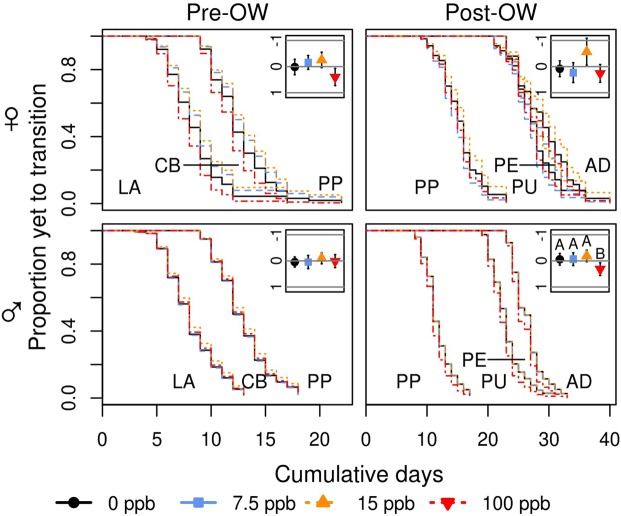
Effect of realistic soil concentrations of imidacloprid on *Megachile rotundata* development speed. Curves represent the transition from one development stage to the next. For example, the group of curves between LA and CB for pre-overwintering male bees represent the transition from a larva to a cocoon-building larva under each treatment. Inset graphs depict the log hazard ratios ±95% confidence intervals (y-axis) associated with each imidacloprid treatment level (x-axis). Values above the centre line (i.e. more negative) represent a lower probability that an individual will transition to the next stage on a given day, provided that it has not already done so, relative to the overall mean. This would result in longer development time. Values below the centerline represent the opposite. Capital letters are used to indicate significant differences (P < 0.05). LA: larva; CB: cocoon-building larva; PP: pre-pupa; PU: pupa; PE: pre-emergent adult; AD: adult; OW: overwintering period.

**Figure 3:**
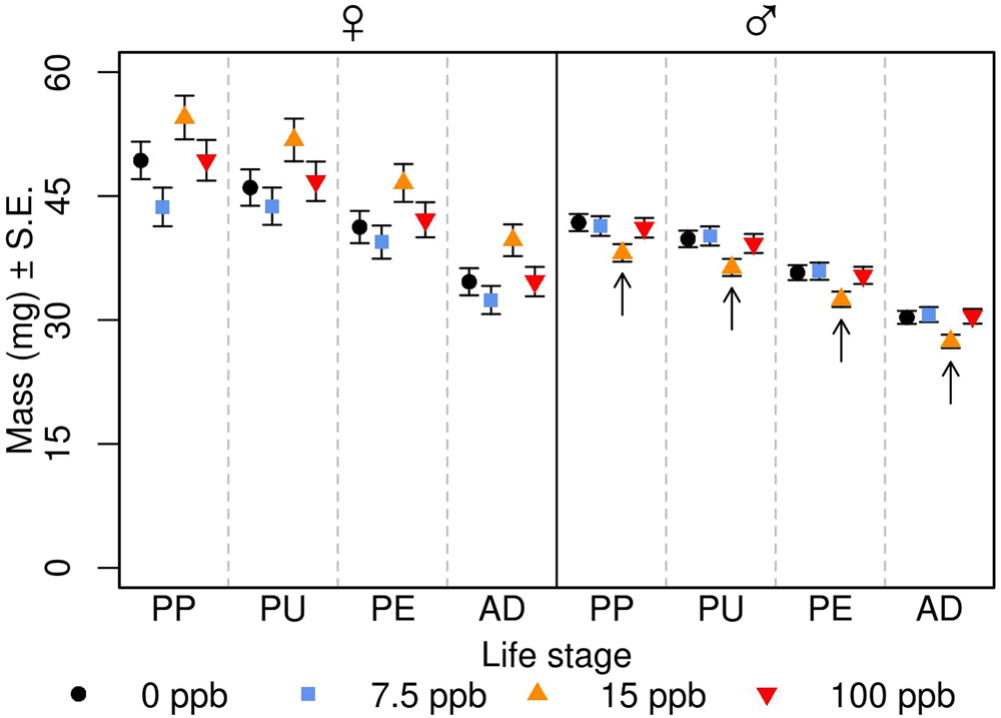
Effect of realistic soil concentrations of imidacloprid on *Megachile rotundata* mass. We included the initial natal cell mass as a covariate and the mass of the shed cocoon in the adult mass. Arrows indicate a treatment level that was significantly different from all other levels at that life stages (P < 0.05). PP: pre-pupa; PU: pupa; PE: pre-emergent adult; AD: adult.

While not statistically significant in comparison with our *a priori* α, there were strong trends for inverted u-shaped effects on female *M*. *rotundata* development speed and mass (Figs 1–3 and S1; Tables 2–4). Individuals treated with 15 ppb developed 1–3 days more slowly in both the pre- and post-overwintering period and weighed 11–20% more than the other treatment levels. Female *M*. *rotundata* treated with 100 ppb developed approximately 2 days faster than control bees during the pre-overwintering stage.

Chronic contact exposure to imidacloprid significantly decreased male *M*. *rotundata* adult longevity, increased post-overwintering development speed, and had a u-shaped effect on mass (Figs 1–3 and S1; Tables 2–4). Male bees treated with the 15 and 100 ppb imidacloprid solutions during development lived 3 and 4 days longer as adults compared to control bees (P = 0.040, P = 0.007, respectively) and there was a trend suggesting that individuals in the 100 ppb treatment lived 2.5 days longer than those in the 7.5 ppb group (P = 0.072). During the post-overwintering period, males in the 100 ppb group developed 1–2 days faster than those in the 0, 7.5, and 15 ppb groups (P = 0.018, P = 0.039, P = 0.010, respectively). Male *M*. *rotundata* treated with the 15 ppb imidacloprid solution were 9% lighter than those treated with 0, 7.5, and 100 ppb (P = 0.013, P = 0.017, P = 0.037, respectively).

## Discussion

The results of this study suggest there are multiple ways imidacloprid contaminated soils can affect bees. Chronic contact exposure in *O*. *lignaria* and *M*. *rotundata* resulted in species- and sex-specific effects on adult longevity, immature development speed, and mass that could have negative consequences for bees more generally. In the case of *O*. *lignaria*, the main effect was decreased adult female longevity at high concentrations of imidacloprid. For *M*. *rotundata*, males responded to increasing imidacloprid exposure with a significant increase in adult longevity and development speed and a u-shaped response in mass. For females, there were strong trends for inverted u-shaped effects on development speed and mass. Species- and sex-specific variation in the effects of imidacloprid on bees have been reported in other studies, reviewed in^1,37^, and could be the result of differing body sizes^38^, life histories (which impacted the number of imidacloprid applications, *see Methods*), genetic differences^17,39^, or number of chromosomes^40^. Despite these limitations, this study demonstrates the potential for neonicotinoid contaminated soils to affect bees.

When exposed to imidacloprid at soil concentrations, we often found biphasic hormetic patterns where bees had opposite responses at intermediate and high concentrations. Hormetic responses are thought to occur when organisms compensate for the negative effects of a stressor at low intensities, often at the expense of other processes, but are unable to keep up at higher intensities^41,42^. Reports of hormetic effects of neonicotinoids, including imidacloprid, are not uncommon for insects^43–46^; however, the underlying mechanisms are unknown. Extrapolating from the results of Derecka *et al*.^34^ and De Smet *et al*.^36^ about the effects of imidacloprid ingestion on honeybee gene expression, the hormetic responses observed here for *M*. *rotundata* development speed and mass may be due to increased expression of detoxification and cuticular protein genes and decreased expression of genes that regulate development. If increased expression of detoxification or cuticular protein genes diverts energy away from other processes^34,36^, then bees would be expected to develop slower or have lower mass. At higher concentrations of imidacloprid, upregulating these genes may be inadequate, and additional strategies are needed such as increased development speed, possibly by decreasing Hsp90 expression^34^, in order to reach the pre-emergent adult stage and the associated thicker cuticle. While this hypothesis explains many of the sub-lethal effects we observed, the insect nervous and endocrine systems are intricately connected and further research is necessary before reaching conclusions about the processes underlying the connection between neurotoxic neonicotinoids and bee development.

In addition to uncertainties about the mechanisms behind sub-lethal effects of imidacloprid on bees, many questions remain about the generalizability across bee species due to genetics and the properties of nest cell linings. While there are ground-nesting *Osmia* and *Megachile*, ground-nesting bees are spread across all seven bee families. If the effects of neonicotinoids can vary based on honeybee genotype^17,39^, it seems likely that responses will vary across Anthophila. Further research on the effects of neonicotinoids across a broader range of taxa will allow us to describe this variability and better predict species’ reactions. Additionally, the effect of nest cell linings on the amount of contact bees have with contaminated soil is unknown. Nest cell linings, commonly secreted from the Dufour’s gland, consist of hydrophobic compounds^47^ and are thought to help maintain moisture homeostasis in brood cells^48^. However, there is great variation between and within species in the use and structure of linings^28–33^ and these barriers may be more permeable than commonly thought as water is hypothesised to cross through the lining into the nest cell^49^. The bee toxicology literature would be greatly enriched by the development of assays to elucidate the permeability characteristics of nest cell linings and to determine which, if any, soil contaminants can cross these barriers.

Although there are reasons to be cautious in applying our results to other bees, the effects observed here suggest that imidacloprid exposure, even at concentrations corresponding to fields 1 to 2 years after treatment^14,16,17^ such as 7.5 and 15 ppb, can have consequences for bee development and survival. Bees nesting in these soils may have increased male or female adult longevity, decreased female development speed, increased female mass, or decreased male mass. While increased longevity and female mass could have a positive effect on bee populations - by increasing the number of cells provisioned and flight ability^50–52^ - reduced energy expenditures as a result of impaired foraging behaviors^10,53,54^ could cause a similar pattern in body mass and would reduce fecundity. If prolonged contact exposure decreases sperm quality in ground-nesting bees like oral exposure does in honeybee drones^8^ or if reduced mass affects male quality in other ways, then increased male longevity may negatively impact bee populations. By living longer, low-quality males could mate with more females, reducing the number of successful fertilisations and driving more male-biased sex ratios. Such an effect would be particularly problematic for individuals or species that only mate once or a few times. In areas with current or long-term imidacloprid use, effects similar to those observed in our 100 ppb treatment may be more common^14,16^. Because the number of offspring produced depends, in part, on adult female longevity, one of the biggest threats to populations in these areas is reduced adult female longevity as observed for *O*. *lignaria*. Further, increased male adult longevity or earlier emergence could intensify the population-level effects described for males in areas with 7.5 to 15 ppb imidacloprid. Further investigations into how neonicotinoid contaminated soils impact bee populations may help elucidate the relationship between our results and the decreases in native bee populations in agricultural landscapes reported elsewhere^12^.

Our results, along with the knowledge that bees are unable to detect neonicotinoids via their olfactory senses and show a preference for contaminated food sources^55,56^, suggests that chronic contact exposure to realistic soil concentrations of neonicotinoids represent a potentially important route of exposure for ground-nesting bees. With the primary approach to bee conservation being the conversion of agricultural fields and adjacent lands into flower-rich habitats^57–60^, caution is advisable in landscapes with a history of neonicotinoid use. If the effects observed here persist in the field, these areas might become ecological traps that lure bees to apparently good resources but actually serve as demographic sinks^61^. Additionally, while the responses of bees to specific neonicotinoids may differ, reviewed in^1,62^, pesticide contamination profiles are likely more complex than a single compound and contaminants may interact in complex ways to strengthen adverse effects on ground-nesting bees. This emphasises the importance of considering and evaluating the effects of chronic contact exposure during development on ground-nesting bee populations in order to better inform responsible pesticide use and to maximise the effectiveness of bee conservation strategies.

## Methods

In order to accommodate differences between *M*. *rotundata* and *O*. *lignaria*, we modified the protocol for each species. These differences and changes are summarised in Table 1 and will be referenced when pertinent in the following description.

### Immature treatment with imidacloprid

We purchased wild-caught, newly laid eggs and early instar larvae from Crown Bees (Seattle, WA) during the spring and summer of 2015. In total, 295 *O*. *lignaria* and 233 *M*. *rotundata* were used for this study (see Table S1 for detailed sample sizes). Individual bees and their pollen provisions were weighed together and placed into a well of a tissue culture plate (Table 1c). Individuals from the same nest were stratified across the treatments to limit the potential genetic biases that exist when evaluating responses to imidacloprid^17,39^. Once individuals reached the second instar larval stage, they were treated every 48 hours with 0.5 μL of 0, 7.5, 15, or 100 ppb imidacloprid (Sigma-Aldrich, PN 37894) in saline solution (Equate Sterile Multipurpose Solution, PN 68113173188) applied topically to their abdominal segments. These concentrations reflect realistic soil concentrations previously reported elsewhere^14,16,17^. Saline solution was used as the solvent because it is less detrimental to larval bees than deionised water (Craig Huntzinger, *personal communication*). Imidacloprid solutions were replaced every 96 hours and kept in the dark at room temperature. In order to maintain a consistent temperature and prevent desiccation, tissue culture plates were kept inside an unheated incubator at room temperature with a 250 mL beaker filled with water. During this time, the chamber temperature was 23.6 ± 0.6 °C and the relative humidity was 84.5 ± 1.3%.

We monitored bee survival and development daily and measured bee mass at important life stages: initial natal nest cell mass (egg and pollen provision), prepupa, pupa, pre-emergent adult, and emergent adult. Shed cocoon mass was added to emergent adult mass to isolate changes due to bee body mass. Tissue culture plates were left open until individuals began spinning cocoons. At that time, lids were replaced to aid in cocoon completion. Once bees constructed cocoons, development was monitored by back-lighting through individual cocoons using a LED light while observing through a stereomicroscope. In October, surviving individuals in their overwintering stages were stored at 4 °C to overwinter. During this time, we placed the tissue culture plates in 53 L plastic tote containers with a 250 mL beaker filled with water to prevent individuals from desiccating. Bees were checked twice a week to ensure humidity was appropriate and to monitor for mould growth. There were no visible signs of mould growth for either species.

In the spring of 2016, bees were removed from cold storage and allowed to emerge (*O*. *lignaria*) or finish their development (*M*. *rotundata*). During this period, we reared *M*. *rotundata* at 28.2 ± 0.1 °C and 78.9 ± 1.8% relative humidity. In order to keep the number of imidacloprid solution applications consistent across individuals of the same species, treatment was stopped after the first individual emerged as an adult. This resulted in zero applications for *O*. *lignaria* and nine for *M*. *rotundata* in 2016 (Table 1e).

### Effects on adult longevity

After emergence, each adult was given a unique paint identifier on the thorax using acrylic paint (Royal Langnickel ACR12). The paint was periodically checked and reapplied as necessary (i.e. if it was damaged or partially missing). For painting, bees were temporarily anaesthetised either by chilling (*O*. *lignaria*) or with carbon dioxide (*M*. *rotundata*). *Megachile rotundata* are less cold tolerant (Tim Krogh, *personal communication*) so they required a modified methodology to prevent undue stress.

Adult bees were placed in 85 L plastic tote containers separated by treatment and species. We provided each treatment group with a flower array, four flowers provided *Typha sp*. pollen, two provided a 2.0 M sucrose solution, and two provided a 1.0 M sucrose solution. Every four days the colour, location within the array, sucrose concentration, and essential oil (*Eugenia caryophyllata*, *Mentha spicata*, *Gaultheria procumbens*, and *Cymbopogon flexuosus*) used in the artificial flowers was randomised to mimic changing resource availability. Similar diets have been provided for other lab cultured bees with success (Emily Dobbs, *personal communication*)^63,64^. We also provided nesting tubes, nesting substrates (Table 1d), and water and replenished these resources as needed. However, no nest cells were completed.

Adult foraging containers were kept in an environmental chamber with a 14:10 light:dark cycle to mimic the daylight patterns of late spring and early summer in Illinois (Philips 32 Watt Alto II PN F32T8/ADV835). The temperature of the environmental chamber was set to 24 °C for *O*. *lignaria* and 28 °C for *M*. *rotundata*. We assessed adult bee mortality and removed deceased individuals daily.

### Statistics

Due to the differences in the number of treatments and total imidacloprid applied (Table 1e–f), *O*. *lignaria* and *M*. *rotundata* were analysed separately. We pooled across sexes for analysis of larval longevity, but otherwise analyzed male and female bees separately. Except where noted, *α* = 0.05 was used to determine statistical significance.

Immature and adult longevity were analysed using Cox Proportional-Hazards Regression^65^ using the ‘rms’^66^ and ‘survival’^67^ packages in the statistical program R^68^. The proportional hazards assumption, checked with the “correlation with time” test described by Harrell^69^, was met for all longevity models (P > 0.096). When there was a significant effect of imidacloprid on longevity, we used Fischer’s LSD contrasts for post-hoc analysis.

Differences in development speed (i.e. the number of days to life events) was analyzed using the Prentice, Williams, and Peterson^70^ total time extension for multiple events (PWP-TT) of the Cox Proportional-Hazards Regression model using the ‘rms’ and ‘survival’ packages in the statistical program R. We set ‘events’ as the transition points between important life stages: larva to cocoon building larva, cocoon building larva to prepupa, prepupa to pupa, pupa to pre-emergent adult, and pre-emergent adult to emergent adult. Separate models were used for the pre- and post-overwintering periods. We censored bees that died during the experiment on their last day of known activity (e.g., movement). The date on which individual bees began treatment with one of the imidacloprid solutions – termed “treatment start date” here *–* was included as a covariate in development speed models and had a significant effect (P < 0.001) in all models except for pre-overwintering *M*. *rotundata* females where it was subsequently removed (χ^2^_1_ = 0.01, P = 0.928). Increased development speed of bees laid later in the season (i.e. those with a later treatment start date) reflects a naturally occurring, yet not completely understood, phenomena^71^. All development models met the assumption of proportional hazards (P > 0.295). Post-hoc analyses using Fisher’s LSD contrast were conducted when there was a significant effect of imidacloprid on development speed.

Bee mass was analysed using linear mixed-effects models with first-order antedependence covariance structures to account for correlation between measurements taken from the same individual at unequally spaced time points in the MIXED procedure in SAS 9.4. We included initial natal cell mass as a covariate in the mass models as final adult size is known to be strongly correlated with pollen provision size^72,73^. This factor was significant in all mass models (F > 9.01, P < 0.004). In order to better meet the assumption of normality, outliers were identified by looking at the Q-Q plots of the residuals and removing extreme values identified by the ROBUSTSCALE option within the UNIVARIATE procedure in SAS 9.4. This approach resulted in dropping one female and three male *O*. *lignaria*. Because linear mixed-effects models are robust against mild departures from the normality assumption, we used an α = 0.025 for Shapiro-Wilk tests of normality. After removing extreme values, the residuals of the linear mixed-effects models were normally distributed (P > 0.034). We used Fisher’s LSD contrasts for post-hoc analyses when we detected a significant effect of imidacloprid concentration in the full models.

## Supplementary information

Supplemental Information

## Data Availability

Data is available through the Illinois Data Bank (10.13012/B2IDB-9033534_V1) and by contacting the corresponding author (N.L.A. nlndrsn2@illinois.edu).
