# Supplementary material for: Chronic contact with realistic soil concentrations of imidacloprid affects the mass, immature development speed, and adult longevity of solitary bees
Source: Sci Rep. 2019 Mar 6;9:3724. doi: 10.1038/s41598-019-40031-9 (PMC6403430; doi:10.1038/s41598-019-40031-9)
Supplement: Supplementary file 1 — Supplemental Information [file 41598_2019_40031_MOESM1_ESM.docx]

**Chronic contact with realistic soil concentrations of imidacloprid affects the mass, immature development speed, and adult longevity of solitary bees**

Nicholas L. Anderson and Alexandra N. Harmon-Threatt

Supplemental Information

**
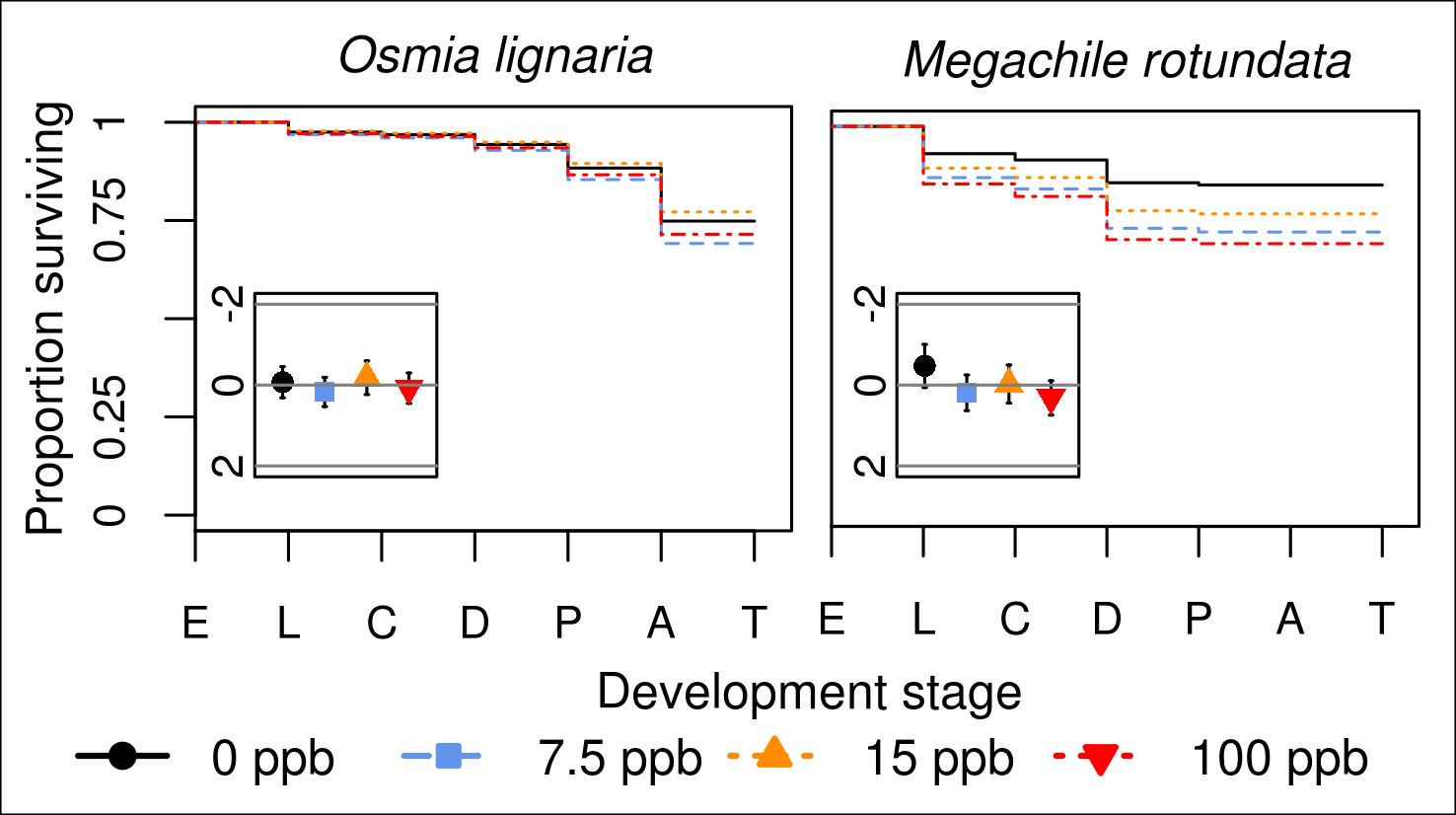
**

**Figure S1. Effect of realistic soil concentrations of imidacloprid on immature bee longevity.** Survival curves represent the proportion of bees that were alive at a given development stage. Inset graphs display the log hazard ratios ± 95% confidence intervals (y-axis) associated with each imidacloprid treatment level (x-axis). The line at zero represents the mean log hazard ratio for that model and values below this line (i.e. more positive) indicate a higher probability that an individual will die on a given day, provided that it has not previously done so, relative to the overall model mean. Values above this line indicate the opposite. E: egg; L: larva; C: cocoon-building larva; D: pre-pupa; P: pupa; A: pre-emergent adult; T: total immature survival (not included in the model).


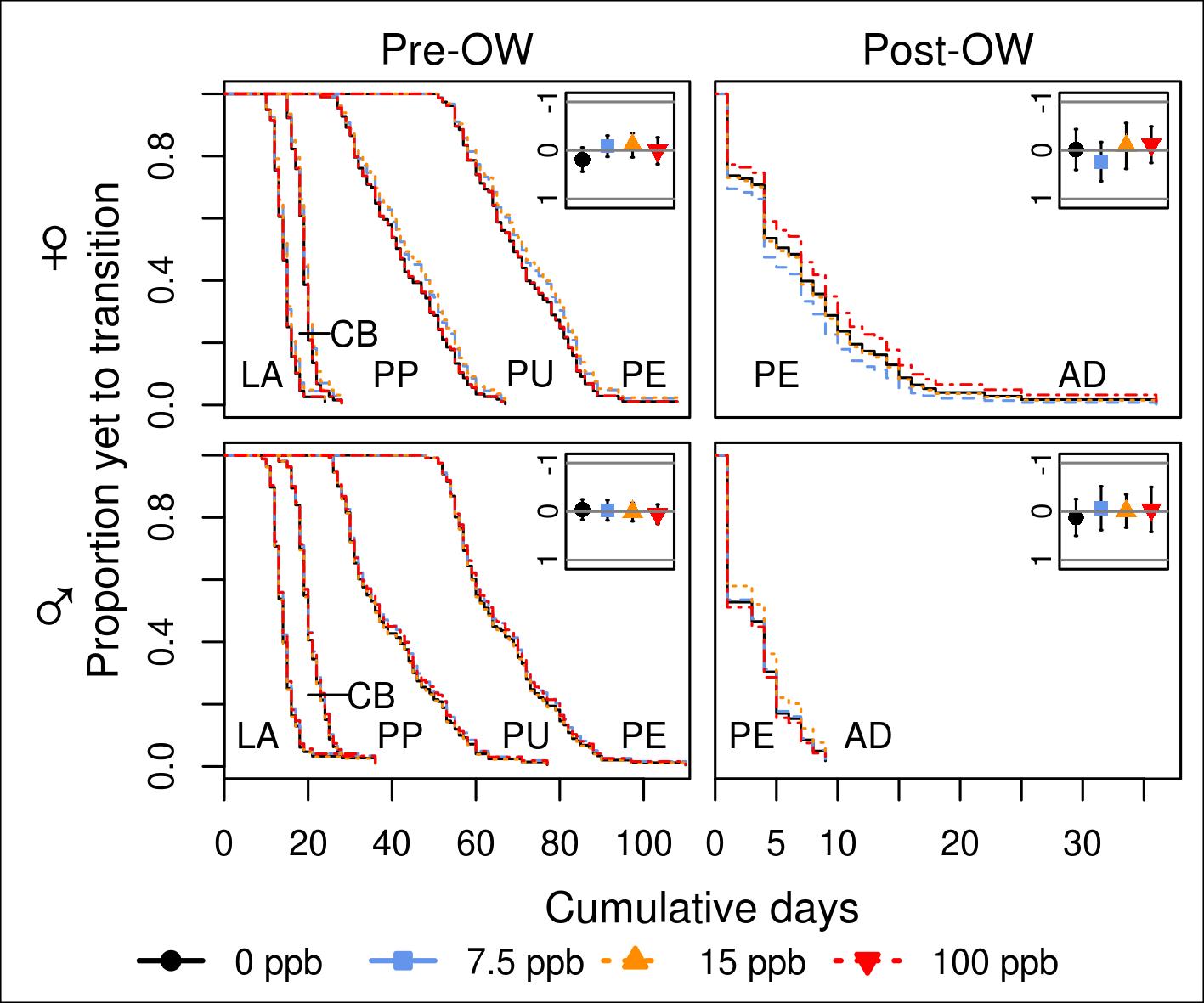


**Figure S2. Effect of realistic soil concentrations of imidacloprid on *Osmia lignaria* development speed.** Curves represent the transition from one development stage to the next. For example, the group of curves between LA and CB for pre-overwintering female bees represent the transition from a larva to a cocoon-building larva under each treatment. Inset graphs depict the log hazard ratios ± 95% confidence intervals (y-axis) associated with each imidacloprid treatment level (x-axis). Values above the centre line (i.e. more negative) represent a lower probability that an individual will transition to the next stage on a given day, provided that it has not already done so, relative to the overall mean. This would result in longer development time. Values below the centerline represent the opposite. LA: larva; CB: cocoon-building larva; PP: pre-pupa; PU: pupa; PE: pre-emergent adult; AD: adult; OW: overwintering period.


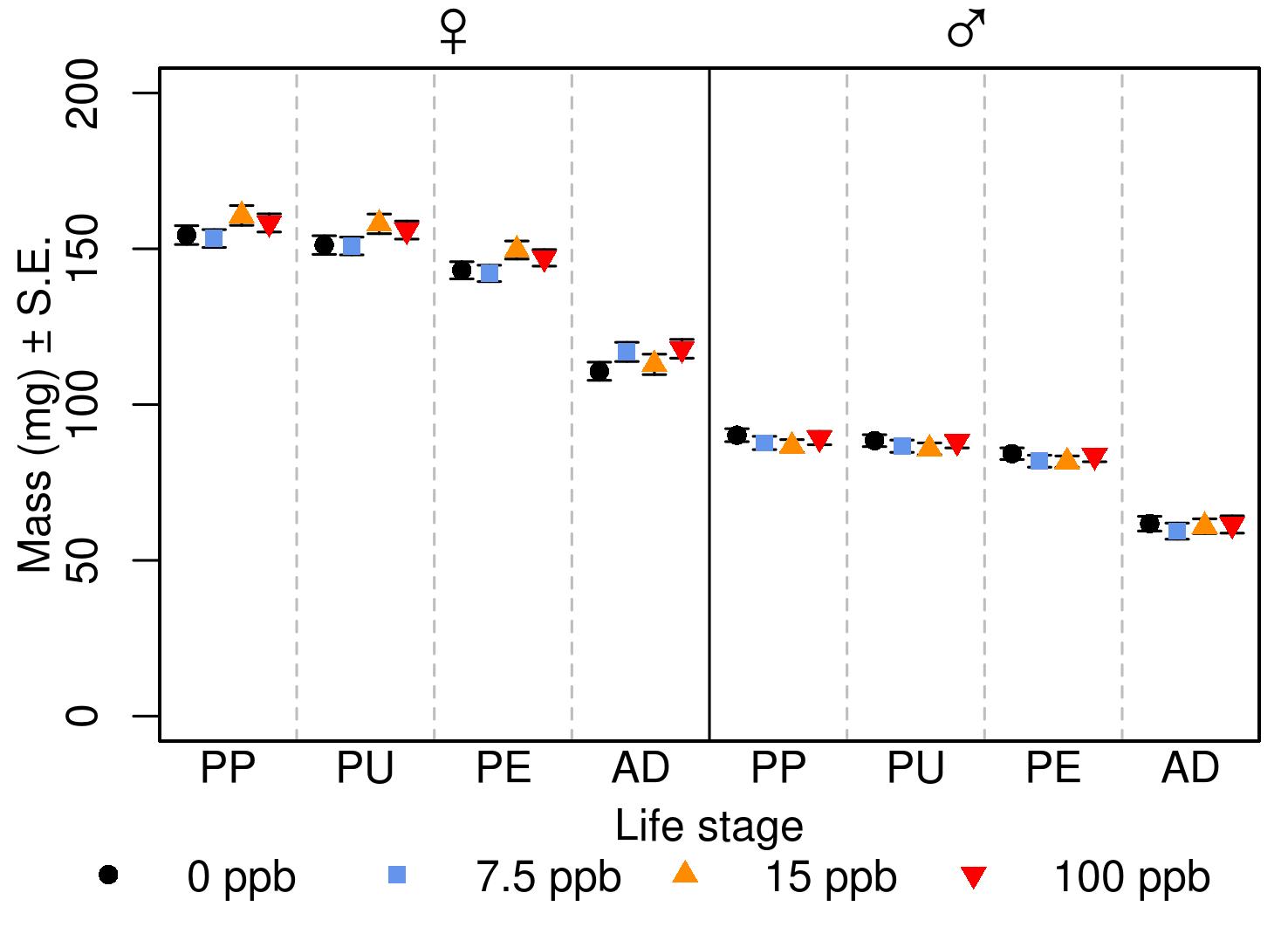


**Figure S3. Effect of realistic soil concentrations of imidacloprid on *Osmia lignaria* mass.** We included the initial natal cell mass as a covariate and the mass of the shed cocoon in the adult mass. PP: pre-pupa; PU: pupa; PE: pre-emergent adult; AD: adult.

**Table S1. Detailed sample sizes.** The number of female and male bees, as well as those whose sex was unknown, present at the start of each life stage. Differences in numbers of bees between life stages represent mortality. *Numbers in parentheses represent bees that emerged early during the overwintering period when there was a mechanical failure. We removed these bees from analyses of adult longevity and body mass.

|  | | ***Osmia lignaria*** | | | | ***Megachile rotundata*** | | | |
| --- | --- | --- | --- | --- | --- | --- | --- | --- | --- |
| **Life Stage** | **Treatment (ppb)** | **Unknown** | **Female** | **Male** | **Total** | **Unknown** | **Female** | **Male** | **Total** |
| **Larvae** | **0** | 2 | 30 | 43 | 75 | 8 | 18 | 33 | 59 |
|  | **7.5** | 2 | 33 | 40 | 75 | 14 | 17 | 27 | 58 |
|  | **15** | 0 | 31 | 41 | 72 | 12 | 13 | 33 | 58 |
|  | **100** | 2 | 32 | 39 | 73 | 17 | 15 | 26 | 58 |
| **Cocoon Building Larvae** | **0** | 2 | 30 | 42 | 74 | 4 | 18 | 33 | 55 |
|  | **7.5** | 2 | 33 | 39 | 74 | 5 | 17 | 27 | 49 |
|  | **15** | 0 | 28 | 40 | 68 | 4 | 12 | 33 | 49 |
|  | **100** | 2 | 32 | 37 | 71 | 11 | 15 | 26 | 52 |
| **Prepupae** | **0** | 1 | 30 | 42 | 73 | 3 | 18 | 33 | 54 |
|  | **7.5** | 2 | 33 | 39 | 74 | 5 | 17 | 27 | 49 |
|  | **15** | 0 | 28 | 40 | 68 | 3 | 13 | 33 | 49 |
|  | **100** | 1 | 32 | 37 | 70 | 7 | 15 | 26 | 48 |
| **Pupae** | **0** | 1 | 30 | 40 | 71 | 0 | 18 | 32 | 50 |
|  | **7.5** | 2 | 32 | 37 | 71 | 0 | 17 | 27 | 44 |
|  | **15** | 0 | 28 | 40 | 68 | 0 | 12 | 33 | 45 |
|  | **100** | 1 | 31 | 35 | 67 | 0 | 14 | 26 | 40 |
| **Pre-emergent Adults** | **0** | - | 28 | 38 | 66 | - | 18 | 32 | 50 |
|  | **7.5** | - | 28 | 34 | 62 | - | 15 | 27 | 42 |
|  | **15** | - | 25 | 37 | 62 | - | 12 | 33 | 45 |
|  | **100** | - | 31 | 34 | 65 | - | 14 | 26 | 40 |
| **Emergent Adults** | **0** | - | 22 (6)* | 7 (21)* | 29 (27)* | - | 18 | 32 | 50 |
|  | **7.5** | - | 17 (10)* | 9 (15)* | 26 (25)* | - | 15 | 26 | 41 |
|  | **15** | - | 16 (6)* | 9 (16)* | 25 (22)* | - | 12 | 32 | 44 |
|  | **100** | - | 20 (10)* | 5 (14)* | 25 (24)* | - | 14 | 26 | 40 |
